# Translation and validation of the Finnish version of index of dental anxiety and fear (IDAF-4C^+^) among dental students

**DOI:** 10.1186/s12903-017-0375-4

**Published:** 2017-05-19

**Authors:** Mimmi Tolvanen, Katri Puijola, Jason M. Armfield, Satu Lahti

**Affiliations:** 1 Department of Community Dentistry, Institute of Dentistry, FI-20014 University of Turku, Turku, Finland; 2 FinnBrain Study Group, Department of Psychiatry and Turku Brain and Mind Center, University of Turku, Turku, Finland; 3 Australian Research Centre for Population Oral Health, Adelaide Dental School, University of Adelaide, Adelaide, 5005 Australia; 4 Turku Clinical Research Center, Turku University Hospital, Turku, Finland

**Keywords:** Dental fear, Dental anxiety, Psychometrics, Validity, Reliability

## Abstract

**Background:**

Dental fear accounts for 41% of the non-habitual dental attendance such as visiting only when in pain among adult Finns. Dentists should be able to recognize patients in risk for irregular attendance due to dental fear and measure their fear with valid and reliable instrument that capture the multidimensionality of dental fear. The study’s aim was to translate the Index of Dental Anxiety and Fear (IDAF-4C+) into Finnish and test its reliability and validity.

**Methods:**

The study population consisted of dental students in a Finnish university (*n* = 202). The IDAF-4C^+^ was back-and forward translated by experts as well as a native English translator, blinded to the original version. Reliability was assessed using Cronbach’s alpha. Validity of the IDAF-4C^+^ was assessed against the Modified Dental Anxiety Scale (MDAS) using Spearman correlation coefficients and through the use of Exploratory factor analysis (EFA) and between genders using Mann-Whitney U tests.

**Results:**

The reliability of the IDAF-4C^+^ was good, the Cronbach’s alpha being 0.88. The IDAF-4C^+^ and MDAS and their subscales were correlated, with coefficients varying between 0.34 and 0.85. Correlations were stronger with the emotional and physiological components of the IDAF-4C^+^. EFA revealed one factor explaining 51.7% of the common variance (eigenvalue = 4.6). Women tended to have slightly higher mean scores than men (1.49 vs. 1.36, *p* = 0.247).

**Conclusions:**

The translation and localization of the Finnish version of the IDAF-4C^+^ can be considered as providing some evidence of the validity and reliability of the scale. It adds to previously used measures as it considers also the behavioral, cognitive and physiological dimension involved in dental fear.

## Background

Dental fear has been found to account for 41% of the non-habitual dental attendance such as visiting only when in pain among adults in Finland [1]. It is therefore important that dentists both recognize patients in risk for irregular attendance due to dental fear and measure their fear with valid and reliable instruments. Measuring dental fear may decrease patient anxiety and is an important step in assisting dentists to manage anxious patients [2, 3]. To this end, a variety of instruments both single and multi-item have been developed to measure dental fear [4–7]. Of these dental fear scales, the Modified Dental Anxiety Scale (MDAS) and a single-item question have so far been validated for adult Finns [8, 9].

The Index of Dental Anxiety and Fear (IDAF-4C^+^) is one of the latest dental anxiety and fear measures [6]. It is comprised of three modules that measure dental anxiety and fear (the core fear module IDAF-4C^+^), dental phobia (the phobia module IDAF-P), and feared dental stimuli (the stimulus module IDAF-S). The specifier 4C is for the four components of fear: emotional, behavioural, physiological, and cognitive and ^+^ indicates the added phobia and stimulus modules. The modular approach makes it possible for clinicians and researchers to use the modules of interest or relevance to them. The core module assesses the general level of dental anxiety and fear whereas the phobia module can be used where a preliminary diagnosis of specific phobia is desired. The stimulus module provides information about the elements of an individual’s dental fear.

The IDAF-4C^+^ differs from other dental fear measures, such as the commonly used MDAS, by highlighting the multidimensional nature of the anxiety and fear construct. While a number of conceptualizations have been used in the literature, Westermeyer [10] has proposed that there are four symptom groups making up a fear response, and these are captured in the four components of the IDAF-4C. To date, however, few scales have used a theoretical basis for the measurement of dental fear [11]. The only other scale designed to assess the multicomponent nature of dental anxiety and fear, the Dental Anxiety Inventory [12], is unwieldy due to the large number of items in the scale, and has been only rarely used. Sufficient evidence is provided to demonstrate that the IDAF-4C^+^ is a useful tool to assess dental fear and anxiety in adult populations and in different languages [6, 13–17]. However, the IDAF-4C^+^ has not yet been translated and validated in Finnish. Thus, the aim of this study was to translate the IDAF-4C^+^ into Finnish and test the reliability and validity in relation to the MDAS.

## Methods

### Translation

Two forward translations were made of the original English IDAF-4C^+^ into Finnish. One translator was aware of the concept and the other, the so-called naive translator, was neither aware nor informed of the concept. A new Finnish version was subsequently derived by SL based on the original and the two translated versions. After that, the native English translator, blinded to the original version, translated it back into the original language as a type of validity check. Finally, an expert committee on dental fear (SL and JA) reviewed all the translations including the original version and reached a consensus on discrepancies.

### Sample

The Finnish version of the IDAF-4C^+^ was tested in the University of Turku and the study population consisted of dental students of all study grades (*n* = 202). A printed questionnaire consisting of three parts was given to every dental student after an exam. The first part was the Finnish translation of the IDAF-4C^+^ consisting of 23 items related to all three modules (8 items for the IDAF-4C, 5 items for the IDAF-P, 10 items for the IDAF-S). The second part was the Finnish version of the MDAS consisting of five questions. Third part consisted of questions about participant’s demographic profile including state of study course, age and gender.

### Measures

The core fear module (IDAF-4C) of the IDAF-4C^+^ consists of eight items with five possible responses to each question, ranging from “disagree” (score = 1) to “strongly agree” (score = 5). The core fear module measures the four components of dental anxiety and fear and contains two items about each component: physiological (“I feel anxious shortly before going to the dentist” and “My heart beats faster when I go to the dentist”), behavioral (“I delay making appointments to go to the dentist” and “I generally avoid going to the dentist because I find the experience unpleasant or distressing”), cognitive (“I think that something really bad would happen to me if I were to visit a dentist” and “I often think about all the things that might go wrong prior to going to the dentist”) and emotional (“I get nervous or edgy about upcoming dental visits” and “I feel afraid or fearful when visiting the dentist”. The scale contains items measuring both anxiety (generally regarded as comprising an anticipatory response) and fear (which comprises the set of responses arising out of actual exposure to the feared stimulus). Those with an IDAF-4C^+^ mean score ≤2.5 were considered to have no to moderate fear, those with mean score ≥3.5 were considered to have high to extreme fear, and those in the middle were considered as having moderate to high fear [7, 15].

The phobia module (IDAF-P) consists of five items with two possible responses, “Yes” or “No”. The first three items address criteria for a dental phobia diagnosis, and the other two items aim to provide a differential diagnosis of social phobia and panic disorder according to the Diagnostic and Statistical Manual of Mental Disorders (DSM-V) [18]. A response of “Yes” to the first three items and a response of “No” to the last two items, together with marked fear according to IDAF-4C^+^ mean score (suggested cut-point mean value ≥ 3), is considered as satisfying a case definition for dental phobia [7, 15].

The stimulus module (IDAF-S) includes 10 items with five possible responses to each question, ranging from “Not at all” (score = 1) to “Very much” (score = 5). These items are intended to be analyzed individually.

The MDAS is a widely used five-item instrument for self-rating dental fear showing validity evidence and high internal consistency (Cronbach’s alpha = 0.93) and reliability over time (intra-class correlation coefficient = 0.93) [19]. The items ask people how they would feel if they “…went to your dentist for treatment tomorrow”, “…were sitting in the waiting room (waiting for treatment)”, “…were about to have a tooth drilled”, “…were about to have your teeth scaled and polished”, and “…were about to have a local anesthetic injection in your gum, above an upper back tooth”. Each item has five response alternatives ranging from 1 (‘Not anxious’) to 5 (‘Extremely anxious’), with the range for total sum score being 5–25. Consistent with previous research, the MDAS total score was categorized into ‘No fear’ (5–9), ‘Low/Moderate fear’ (10–18) and ‘High fear’ (19 or more) groups. MDAS has a two factor structure, consisting of anticipatory anxiety (items 1 and 2, range 2–10) and treatment related anxiety (items 3, 4 and 5, range 3–15) [20].

### Statistical analyses

Distributions of IDAF-4C^+^, IDAF-4P and IDAF-4S items were evaluated. Prevalence estimates were calculated for IDAF-4C^+^ and MDAS using the pre-defined cut-points for both scales. The prevalence estimates were compared by participant gender with both scales using cross tabulation and chi square test. As IDAF-4C+ has previously demonstrated one factor structure exploratory factor analysis (EFA) with varimax rotation was conducted to see if there were more than one underlying constructs within the measure. The differences in IDAF-4C^+^ and MDAS scores between male and female participants were evaluated with Mann-Whitney U tests. The associations between IDAF-4C^+^ and MDAS scores and their components were evaluated with Spearman correlation coefficients. Reliability of the scales was evaluated using Cronbach’s alphas.

## Results

Of the 202 administered questionnaires, 172 were returned, giving a response rate of 85%. The mean age of participants was 23.4 years (range 19 to 33 years old, median = 23, SD = 2.4) and the majority were female (69.2%). Mean values of the IDAF-4C^+^ and MDAS as well as their subscales are presented in Table 1. Both total scores were positively skewed: IDAF-4C^+^ median = 1.25 range = 1–5, Q_1_–Q_2_ = 1.13–1.52 and MDAS median = 8 range = 5–25, Q_1_–Q_2_ = 7–11.

**Table 1:** Descriptive statistics for the IDAF-4C and MDAS by gender (Mann-Whitney U test)

	Total *n* = 172		Female *n* = 117		Male *n* = 52		*p*
	Mean (SD)	Md	Mean (SD)	Md	Mean (SD)	Md	
IDAF-4C total score	1.45 (0.58)	1.3	1.49 (0.63)	1.3	1.36 (0.46)	1.3	0.247
IDAF-4C physiological	1.54 (0.68)	1.5	1.58 (0.72)	1.5	1.47 (0.60)	1.5	0.545
IDAF-4C behavioral	1.41 (0.73)	1.0	1.40 (0.78)	1.0	1.42 (0.61)	1.0	0.195
IDAF-4C cognitive	1.25 (0.54)	1.0	1.29 (0.58)	1.0	1.18 (0.42)	1.0	0.300
IDAF-4C emotional	1.60 (0.80)	1.5	1.71 (0.86)	1.5	1.35 (0.57)	1.0	0.003
MDAS total sum	8.98 (3.01)	8	9.32 (3.16)	9	8.35 (2.55)	8	0.086
MDAS F1 sum	3.16 (1.37)	3	3.22 (1.47)	3	3.10 (1.11)	3	0.986
MDAS F2 sum	5.81 (2.01)	5	6.09 (2.07)	6	5.25 (1.81)	5	0.012

*IDAF-4C* Index of Dental Anxiety and Fear, *MDAS* Modified Dental Anxiety Scale, *F1* Treatment related anxiety factor, *F2* Anticipatory anxiety factor

Using the adopted MDAS cut-points, of the participants 61.6% had no fear; 37.2% had low/moderate dental fear and 1.2% had high dental fear (1.7% of women and 0% of men). According to the IDAF-4C^+^, of the participants 67.4% had no to low fear (score 1–1.49), 24.9% had low to moderate fear (score 1.5–2.49). 6.2% had moderate to high fear, and 1.2% had high to extreme dental fear (1.7% of women and 0% of men). There were no statistically significant gender differences in any of these prevalence estimates.

Exploratory factor analysis of the IDAF-4C^+^ revealed one factor explaining 51.7% of the common variance (eigenvalue = 4.6). With both the IDAF-4C^+^ and MDAS, women tended to have slightly higher mean scores than men (Table 1). However, the differences were statistically significant only for the IDAF-4C^+^ emotional component and the MDAS factor treatment-related anxiety. The IDAF-4C^+^ and MDAS and their subscales were correlated, with coefficients varying between 0.34 and 0.85 (Table 2). The reliability of the scales was good, the Cronbach’s alphas being 0.882 for the IDAF-4C^+^ and 0.789 for the MDAS.

**Table 2:** Spearman correlation coefficients between IDAF-4C and MDAS total scores and components (all *p*-values <0.001)

	IDAF-4C total score	IDAF-4C ^+^physiological	IDAF-4C behavioral	IDAF-4C ^+^cognitive	IDAF-4C ^+^emotional
IDAF-4C physiological	0.85				
IDAF-4C behavioral	0.70	0.53			
IDAF-4C cognitive	0.56	0.42	0.34		
IDAF-4C emotional	0.82	0.63	0.43	0.39	
MDAS total sum	0.74	0.65	0.49	0.40	0.69
MDAS F1 sum	0.72	0.69	0.51	0.38	0.64
MDAS F2 sum	0.63	0.53	0.38	0.35	0.61

*NOTE*: all *p*-values <0.001; *IDAF-4C* Index of Dental Anxiety and Fear, *MDAS* Modified Dental Anxiety Scale, *F1* Treatment related anxiety factor, *F2* Anticipatory anxiety factor

There were no participants who could have been considered as dentally phobic (Table 3). All participants answering “Yes” to any of the IDAF-4P items were women, except for one.

**Table 3:** Number of participants answering “yes” to IDAF-4P (phobia module) items

IDAF-4P items	*n*	%
Going to the dentist is actively avoided or else endured with intense fear or anxiety	5	2.9
My fear of going to the dentist has been present for at least 6 months	10	5.8
My fear, anxiety or avoidance of going to the dentist significantly affects my life in some way	0	0
I am afraid of going to the dentist because I am concerned I may have a panic attack	1	0.6
I am afraid of going to the dentist because I am generally highly self-conscious or concerned about being watched or judged in social situations	5	2.9

Women tended to report higher scores for the IDAF-S items, but the difference was statistically significant only for ‘having an unsympathetic or unkind dentist’, and approached statistical significance for ‘numbness caused by the anesthetic’ and ‘not knowing what dentist is going to do’ (Table 4).

**Table 4:** Mean values for IDAF-S15 (stimulus module) items by gender

IDAF-S items	All	Female	Male	*p*
Painful or uncomfortable procedures	2.37	2.44	2.19	0.145
Feeling embarrassed or ashamed	1.35	1.37	1.31	0.562
Not in control of what is happening	1.62	1.68	1.48	0.269
Feeling sick, queasy or disgusted	1.36	1.36	1.37	0.844
Numbness caused by the anaesthetic	1.47	1.53	1.35	0.051
Not knowing what dentist is going to do	1.61	1.68	1.43	0.085
The cost of dental treatment	1.62	1.68	1.50	0.164
Needles or injections	1.97	2.04	1.81	0.167
Gagging or choking	1.56	1.56	1.56	0.888
Having an unsympathetic or unkind dentist	1.90	2.05	1.56	0.007

## Discussion

The Finnish version of the IDAF-4C^+^ demonstrated both validity and reliability evidence. Dental fear and anxiety were more common among women than men on both the IDAF-4C^+^ and MDAS, though the difference between genders was not statistically significant except in the emotional component of the IDAF-4C^+^ and the treatment-related factor of the MDAS. The number of participants with no dental fear or anxiety was higher with IDAF-4C^+^ than with MDAS, which may be due to somewhat arbitrary/explorative cut-off points which have currently been used in just one or two studies. However, the percentages with high fear were similar. The associations between the scales were strong and the internal consistency of the scales, assessed using Cronbach’s alpha, were good.

Within the IDAF-4C subscales, the correlation between the physiological and the emotional component was the strongest and the correlation between the cognitive and the behavioral component the weakest. This is in line with previous studies about the development of the IDAF-4C^+^ [7] and adaptation of the Spanish and Swedish versions of the IDAF-4C^+^ [15, 17]. The association between the IDAF-4C and MDAS was strongest for full-scale scores. Of the subscales, the IDAF-4C physiological and emotional components had a stronger correlation with the MDAS and its subscales than the behavioral and cognitive components, which is also in line with previous studies, although associations with MDAS factors have not been reported before, only with the MDAS total score [15]. Of the MDAS factors, treatment-related anxiety had stronger correlations with the IDAF-4C than did anticipatory anxiety. The stronger correlation between treatment-related anxiety and the IDAF-4C^+^ is logical as their contents resemble one another more than the contents of anticipatory anxiety and the core dental fear and anxiety module. Like with the MDAS in this study, the Dental Anxiety Scale (DAS) [4], from which MDAS was modified, has been found to be more strongly associated with the physiological and emotional components of the IDAF-4C and had weaker correlations with the behavioral and cognitive components [7]. Even though the DAS and MDAS are different scales, they are suggested to be coaxial [21]. The IDAF-4C has also been reported to have statistically significant correlations with the DAS and a single-item measure of dental fear, but to be better at predicting fear of loss of control, avoidance of the dentist owing to fear and problem-oriented visiting, and to have a higher sensitivity and specificity than the other two scales when using a higher cut-point [14].

The nature of dental fear is multidimensional, which makes it difficult to study [19, 22, 23]. Most dental fear scales have been criticized for being psychometrically insufficient and for lacking a theoretical framework [7, 23]. The MDAS mainly focuses on the emotional response to dental stimuli while the IDAF-4C additionally considers the behavioral, cognitive and physiological dimension involved in dental fear, which makes the IDAF-4C^+^ a more complete measure [15].

When measures are being used across cultures, the items must undergo appropriate linguistic translation and also be adapted culturally to maintain the content validity of the instrument at a conceptual level across the cultures. This is called the cross-cultural adaptation process. The aim is to produce equivalence between source and target base on content. The cross-cultural adaptation process ensures retention of psychometric properties such as validity and reliability at an item and/or a scale level [24]. The process also involves back translation as a type of validity check, highlighting inconsistencies or conceptual errors in the translation [24]. These procedures were followed in our study. It has been shown that it is possible to find consensus on principles of good practice in translation and cultural adaptation by looking for the areas of agreement in broader terms and allowing for different ways to achieve the same goal for each step in the process of translation [25]. This study shows that the Finnish version of IDAF-4C^+^ has evidence of reliability and validity, supporting the argument that the translation process was successful.

While the findings in relation to participant dental anxiety can not be generalized to the general Finnish population, University students have previously been found to be a rich source of potential participants for dental fear research, often demonstrating levels of dental fear and anxiety similar to that found in the general population [26]. The mean values for MDAS scores of women and men were rather similar to those found among pregnant Finnish families [27]. However, prevalence of high dental fear among these healthy University students was lower than among 19–33-year-old Finnish population (8–10% in 2000) [28]. Also in this study, the number of men, especially highly anxious, was significantly lower compared to the number of women. Although the response rate was good in this study, the study population was quite small and presented particular characteristics with respect to gender, age range and educational level. This should be acknowledged when interpreting the results. However, even though validity in terms of gender differences in dental fear could not be observed in this study it is likely that in more representative population with sufficient number of fearful respondents IDAF-4C+ would show better validity in this aspect.

## Conclusions

In conclusion, the translation and localization of this Finnish IDAF-4C^+^ can be considered as providing evidence of the validity and reliability of the scale. Given the strong theoretical foundations of the scale and the results found here, the IDAF-4C^+^ can be recommended for future research endeavors where it is important to measure dental anxiety and also for clinicians wishing to screen patients for dental fear and anxiety in order to better determine a potentially successful treatment plan.

## Abbreviations

DAS: Dental Anxiety Scale
DSM-V: Diagnostic and Statistical Manual of Mental Disorders
EFA: Exploratory factor analysis
F1: Treatment related anxiety factor of MDAS
F2: Anticipatory anxiety factor of MDAS
IDAF-4C^+^: Index of Dental Anxiety and Fear
IDAF-B: The behavioural module IDAF-4C^+^
IDAF-C: The cognitive module IDAF-4C^+^
IDAF-P: The phobia module of IDAF-4C^+^
IDAF-S: The stimulus module IDAF-4C^+^
MDAS: Modified Dental Anxiety Scale
